# A community-engaged approach to understanding environmental health concerns and solutions in urban and rural communities

**DOI:** 10.1186/s12889-021-11799-1

**Published:** 2021-09-24

**Authors:** Suwei Wang, Molly B. Richardson, Mary B. Evans, Ethel Johnson, Sheryl Threadgill-Matthews, Sheila Tyson, Katherine L. White, Julia M. Gohlke

**Affiliations:** 1 Translational Biology, Medicine, and Health Program, Virginia Polytechnic Institute and State University, Blacksburg, VA 24061 USA; 2 Department of Population Health Sciences, VA-MD College of Veterinary Medicine, Virginia Polytechnic Institute and State University, 205 Duck Pond Drive, Blacksburg, VA 24061-0395 USA; 3 Division of Preventive Medicine, School of Medicine, University of Alabama at Birmingham, Birmingham, AL 35233 USA; 4 Center for the Study of Community Health, University of Alabama at Birmingham, Birmingham, AL 35233 USA; 5 West Central Alabama Community Health Improvement League, Camden, AL 36726 USA; 6 Friends of West End, Birmingham, AL 35228 USA

**Keywords:** Community-engaged, Environmental health, Focus group, Workshops, Urban-rural comparison

## Abstract

**Background:**

Focus groups and workshops can be used to gain insights into the persistence of and potential solutions for environmental health priorities in underserved areas. The objective of this study was to characterize focus group and workshop outcomes of a community-academic partnership focused on addressing environmental health priorities in an urban and a rural location in Alabama between 2012 and 2019.

**Methods:**

Six focus groups were conducted in 2016 with 60 participants from the City of Birmingham (urban) and 51 participants from Wilcox County (rural), Alabama to discuss solutions for identified environmental health priorities based on previous focus group results in 2012. Recorded focus groups were transcribed and analyzed using the grounded theory approach. Four follow-up workshops that included written survey instruments were conducted to further explore identified priorities and determine whether the priorities change over time in the same urban (68 participants) and rural (72 participants) locations in 2018 and 2019.

**Results:**

Consistent with focus groups in 2012, all six focus groups in 2016 in Birmingham identified abandoned houses as the primary environmental priority. Four groups listed attending city council meetings, contacting government agencies and reporting issues as individual-level solutions. Identified city-level solutions included city-led confiscation, tearing down and transferring of abandoned property ownership. In Wilcox County, all six groups agreed the top priority was drinking water quality, consistent with results in 2012. While the priority was different in Birmingham versus Wilcox County, the top identified reason for problem persistence was similar, namely unresponsive authorities. Additionally, individual-level solutions identified by Wilcox County focus groups were similar to Birmingham, including contacting and pressuring agencies and developing petitions and protesting to raise awareness, while local policy-level solutions identified in Wilcox County included government-led provision of grants to improve septic systems, and transparency in allocation of funds. Workshops in 2018 and 2019 further emphasized water quality as the top priority in Wilcox County, while participants in Birmingham transitioned from abandoned houses as a top priority in 2018 to drinking water quality as a new priority in 2019.

**Conclusions:**

Applying a community-engaged approach in both urban and rural locations provided better understanding of the unique opportunities and challenges for identifying potential interventions for environmental health priorities in both locations. Results can help inform future efforts to address locally defined environmental health issues and solutions.

**Supplementary Information:**

The online version contains supplementary material available at 10.1186/s12889-021-11799-1.

## Introduction

A healthy environment is essential for improving the quality of life and the extent of healthy living. Worldwide, preventable environmental factors are responsible for 23% of all deaths and 26% of deaths among children less than 5 years old [1]. Environmental factors are diverse with far-reaching impacts on health [2]. Community engaged research in environmental health includes a variety of non-academic stakeholders, such as residents in affected neighborhoods, neighborhood leaders, non-governmental agencies, and government agency representation. It is designed to improve our understanding of environmental factors affecting health that may be the most promising to address based on local priorities and circumstances.

Focus groups are one of the methods for facilitating community-engaged research. It is an invaluable tool for researchers investigating community’s perceptions of environmental hazards, because they provide a setting for gathering resident’s knowledge and establishes common ground among participants and between participants and researchers [3]. Local focus groups can engage residents and identify ways to work towards solving a problem collaboratively [4]. Because of the small size and informal nature of focus groups, participants can build on and debate each other’s responses, which helps to better understand an issue and the influences surrounding it [5]. This understanding and information are obtained in a relatively short amount of time, so focus groups are an efficient way for researchers to attain information [6]. Furthermore, focus groups can enlighten researchers to perceived environmental hazards not previously considered [3]. All of these attributes are essential for developing a feasible, acceptable, and supported intervention to address health outcomes associated with environmental factors [7].

In 2010 we initiated a community-academic partnership, ENACT, between Friends of West End (FoWE) in Birmingham, Alabama (AL), and West Central Alabama Community Health Improvement League (WCACHIL) in Wilcox County, AL and Virginia Tech, University of Alabama at Birmingham, and Johns Hopkins University [8]. Through ENACT we work on environmental health issues in Alabama with successful completion of several environmental epidemiology studies, focus groups, workshops, and phone surveys [9–14]. In our initial focus groups in 2012, we found that abandoned houses was the highest environmental health priority in Birmingham, Alabama while inadequate sewer and water services was the top priority in Wilcox County, Alabama [9]. A follow-up larger scale and randomly sampled phone survey reaffirmed these priorities in each community and furthered our understanding of how resident priorities are similar or different from local health agency priorities [11]. Additionally, follow-up community-engaged research allows the dissemination of updated results and collection of new information to verify previous results and further refine the most appropriate path to mitigate health outcomes associated with environmental health priorities.

Water and sewage service issues and abandoned houses and lots are significant environmental health problems in rural and urban areas in the United States, respectively. Unsewered homes are common in rural areas of the United States, leading to increased risk of a variety of infectious diseases [15]. For example, soil transmitted helminth infections were identified in household members without adequate sewage in rural Alabama [16]. Drinking water service characteristics have also been associated with reported gastrointestinal illness in rural Alabama [17]. Garvin et al. (2013) found abandoned properties affect community well-being via overshadowing positive aspects of community, producing fractures between neighbors, attracting crime, and making residents fearful [18]. Other research found the problem is perceived as particularly widespread in the U.S. South, where our study areas are located [19]. Abandoned houses and lots can contribute to numerous health and safety hazards including falling debris, vermin, mold, standing water, toxic chemicals, and sharp rusty objects [20], and can have negative impacts on housing/neighborhood vitality, violence and crime prevention efforts, fire and vandalism risk, commercial district vitality, and assessed property values, etc. [19, 21–24].

In the present study, we build from our previous findings to 1) better understand why those problems persist, 2) what residents feel the solution is, and who is responsible for enacting the solution 3) identify ways to support residents in identifying a process to address environmental concerns and 4) examine whether environmental health priorities change over time. Applying this community-engaged approach in both an urban and a rural location allowed us to better understand the unique opportunities and challenges in both locations.

## Methods

### Characteristics of focus group study populations

Birmingham, AL is the largest city in Alabama with a population of 209,403, of which 70.5% of the population identifies as Black or African American [25]. Birmingham has a poverty rate of 27.2%, and those identifying as Black or African American comprise 76.9% of those living in poverty [25]. Wilcox County, AL, a rural setting, has a lower population of 10,300, of which 71.3% identify as Black or African American [25]. A total of 33.4% of the population in Wilcox County live in poverty and of that 88.2% identify as Black or African American [25].

### Focus group procedure

This study involved Virginia Tech and University of Alabama at Birmingham researchers collaborating with Friends of West End (FoWE) in Birmingham, AL, and West Central Alabama Community Health Improvement League (WCACHIL) in Wilcox County, AL as part of an ongoing community-academic partnership, ENACT [8]. The protocol was approved by the Virginia Tech Institutional Review Board (15–761). The community partners recruited participants aged at least 18 without regard to sex, ethnicity or ancestry. WCACHIL recruited 51 participants in Wilcox County and FoWE recruited 60 participants in Birmingham using a convenience and snowball sampling approach. The number of focus groups (*N* = 12, 6 in Birmingham city, AL and 6 in Wilcox County, AL) was based on the need to expand our line of questions from our previous focus groups investigating environmental health priorities in 2012 (*N* = 8 in total, 4 in Birmingham, AL and 4 in Wilcox County, AL) [9]. This new direction led us to include two more focus groups in each location, and this number of focus groups is consistent with previous studies exploring data saturation across a wide range of topics [26–29].

Community and academic partners together drafted and agreed upon a guide of questions to ensure appropriateness and consistency between groups. The guide followed a natural progression of identifying positive attributes of participants’ neighborhoods, determining whether previously identified environmental health priorities (abandoned houses and overgrown lots in Birmingham and drinking water access and quality in Wilcox County) [9] were still priority issues, why the problems persisted, who was responsible for solving them, and what participants felt were the solutions to address those priority issues.

We took a positivist approach in focus group data collection and analysis. The facilitator guide (Additional file 1) emphasized encouraging all participants to contribute, embracing new ideas, and enforcing respect for all participants’ comments [30]. Members of the community-academic partnership served as facilitators in each group. Facilitators were encouraged to utilize strong listening and questioning skills, prodding participants with prompts to encourage them to speak up or clarify statements. All facilitators had training in the value of focus groups and the best practices for facilitating focus groups that are partly based on works by Franz et al., Drake et al. [31, 32]*.* Facilitators aimed to document subjects opinions and attitudes in an objective way, assuming a detached, independent role in the discussion, but ensuring focus groups followed the structured guide [33]. Facilitators were provided guidance and practice on how to draw out concerns while not bringing bias by sharing their own views [34–37], drawing adequate participation from each participant, and minimizing the influence of dominant speaker(s) views. In training and planning for focus groups, we placed emphasis on the importance of the role of the facilitator, having groups be of reasonable size (8–10 individuals), and individuals not being too familiar with other group members [33].

Twelve focus groups were conducted in September 2016, six in Birmingham and six in Wilcox County. Focus groups were organized to be at a time when participants would be available and within familiar neighborhood gathering places to increase comfort in active participation. Approximately 10 participants sat at each table with facilitators. Facilitators went through formal Institutional Review Board consent, then initiated recording of the focus group with digital recorders. Focus groups lasted approximately one-hour and participants completed a written survey. Facilitators’ field notes were considered during the coding process, described below.

### Focus group data analysis

Researchers do not editorialize participants’ opinions and remained non-judgmental and respectful [37]. Recordings were transcribed by the second author. In the first stage, transcriptions were coded into categories based on questions posed (determined a priori) from the script independently by second author and third author (Additional file 2). The second author then went through all coded transcripts combining responses identified to be most inclusive [38]. In the event that a statement responded to multiple questions (i.e., responsible parties and solutions), they were coded to each question response. In the next stage, the second author and third/seventh author independently further subcategorized the inclusive coded transcripts per the subcategorization coding tree (Additional file 2). Summaries were then consolidated and presented by focus group and by location with verbatim. Recordings from all focus groups were analyzed and coded to ensure all themes discussed are presented in the results. Interrater reliability was assessed for topics: reasons for persistence, responsible parties, sources of trusted information, and other priorities brought up. Interrater reliability rate (IRR) was high in both Wilcox County transcripts and Birmingham transcripts (IRR = 90.6% in Wilcox County, 91.9% in Birmingham). An IRR of 90.6% reflects that 512 out of 563 responses were categorized the same between the two coders.

### Follow-up workshops

Preliminary results from the focus groups were compiled and used to develop a survey instrument to further explore environmental health priorities and solutions and implemented at workshops in May 2018. Fifty-three participants from the same urban and rural locations (23 in Birmingham and 30 in Wilcox County) attended the workshops and completed the survey. A total of 92 participants from the same urban and rural locations (49 in Birmingham and 43 in Wilcox County) attended another two follow-up workshops in September 2019. A collaborative presentation by researchers and community partners on spatially explicit risk maps developed from our retrospective analysis of adverse health outcomes associated with heatwaves in Alabama was given. Participants filled out a written survey ranking the most concerning environmental health issues. Demographic information was also collected in the survey instruments administered in 2018 and 2019. The agendas for the workshops are shown in Additional file 1. All survey instruments are accessible at our research outreach website [39, 40].

Answers to the surveys were summarized and compared between Birmingham and Wilcox County participants in 2018 and 2019, respectively. The responses to open-ended questions were first coded into categories by the first author, then independently coded by the eighth author using the categories established by the first author. Any differences were discussed to resolve final categorization. The rankings of six environmental health issues were converted to Likert scale, with average ranks computed for ties. For a specific environmental health issue, the Mann-Whitney test was used to determine whether the medians of the ranks were different in Birmingham vs. Wilcox County.

## Results

### Study population

Most participants in the 2016 focus groups (92%) and 2018 (98%) and 2019 (86%) workshops self-identified as Black or African American (Table 1). In 2016 focus groups, urban and rural participants had similar gender ratio (67, 80% female, respectively), education level (48, 53% with higher than high school diploma, respectively), annual household income (68, 55% at <$ 20,000, respectively), and general health (92, 90% responding in good health condition, respectively) while urban participants were older compared to rural participants (mean age 60 in urban vs. 52 in rural, *p*-value 7.9E-03). In 2018 workshops, the only urban-rural difference among participants was that a higher percent of rural participants participated in the 2016 focus groups (63% in rural vs. 26% in urban, *p*-value 0.02). In 2019 workshops, urban participants were younger (mean age 50 in urban vs. 58 in rural, *p*-value 0.02), had a lower percent in annual household income ≥$20,000 (42% in urban vs. 76% in rural, *p-*value 1.6E-04), a lower percent in participation of 2017 monitor study (16% in urban vs. 60% in rural, *p*-value 3.4E-05) and a lower percent in participation of 2016 focus groups (18% in urban vs. 62% in rural, *p*-value 8.8E-05) (Table 1).

**Table 1:** Characteristics of 2016 focus group participants and 2018, 2019 workshop participants

Year	2016			2018			2019		
Location	Birmingham	Wilcox County	*p*-Value^a^	Birmingham	Wilcox County	*p*-Value^a^	Birmingham	Wilcox County	*p*-Value^a^
Number	60	51		23	30		45	42	
Age			7.9E-03*			0.59			2.1E-02*
Mean(95%CI)	60.1 (59.6–60.6)	51.9 (51.3–52.6)		51.0 (49.7–52.2)	53.1 (52.2–54.0)		49.6 (48.6–50.6)	58.0 (57.5–58.5)	
Median(range)	62 (23–85)	56 (20–81)		49 (25–71)	58 (19–69)		60 (19–73)	60.5 (34–73)	
Missing	4 (7%)	2 (4%)		0 (0%)	0 (0%)		6 (13%)	2 (5%)	
Sex			0.11			0.53			0.54
Female	40 (67%)	41 (80%)		22 (96%)	26 (87%)		32 (71%)	35 (83%)	
Male	20 (33%)	9 (18%)		1 (4%)	4 (13%)		8 (18%)	5 (12%)	
Missing	0 (0%)	1 (2%)		0 (0%)	0 (0%)		5 (11%)	2 (5%)	
Race									
African American or Black	56 (93%)	46 (90%)	1.00	23 (100%)	29 (97%)	1.00	38 (84%)	37 (88%)	0.49
Other^b^	3 (5%)	2 (4%)		0 (0%)	1 (3%)		0 (0%)	2 (5%)	
Missing	1 (2%)	3 (6%)		0 (0%)	0 (0%)		7 (16%)	3 (7%)	
Education			0.66			0.83			1.00
≤ High school	29 (48%)	21 (41%)		9 (39%)	10 (33%)		14 (31%)	13 (31%)	
> High school	29 (48%)	27 (53%)		12 (52%)	18 (60%)		24 (53%)	25 (60%)	
Missing	2 (3%)	3 (6%)		2 (9%)	2 (7%)		7 (16%)	4 (10%)	
Income			0.16			0.26			1.6E-04*
< $20,000	41 (68%)	28 (55%)		7 (30%)	15 (50%)		16 (36%)	1 (2%)	
≥ $20,000	12 (20%)	17 (33%)		15 (65%)	14 (47%)		19 (42%)	32 (76%)	
Missing	7 (12%)	6 (12%)		1 (4%)	1 (3%)		10 (22%)	9 (21%)	
Health									1.00
Good^c^	55 (92%)	46 (90%)	1.00	22 (96%)	30 (100%)	NA	37 (82%)	37 (88%)	
Poor	2 (3%)	2 (4%)		0 (0%)	0 (0%)		1 (2%)	1 (2%)	
Missing	3 (5%)	3 (6%)		1 (4%)	0 (0%)		7 (16%)	4 (10%)	
Participant in monitor study (summer 2017)			NA			0.68			3.4E-05*
Yes	NA	NA		16 (66%)	19 (63%)		7 (16%)	25 (60%)	
No	NA	NA		6 (26%)	11 (37%)		32 (71%)	12 (29%)	
Missing	NA	NA		1 (4%)	0 (0%)		6 (13%)	5 (12%)	
Participant in previous focus groups			NA			0.02*			8.8E-05*
Yes	NA	NA		6 (26%)	19 (63%)		8 (18%)	26 (62%)	
No	NA	NA		16 (70%)	10 (33%)		30 (67%)	12 (29%)	
Missing	NA	NA		1 (4%)	1 (3%)		7 (16%)	4 (10%)	
How long they have lived in this community			NA			0.05			0.011*
0-5 years	NA	NA		6 (26%)	1 (3%)		8 (18%)	4 (10%)	
6–15 years	NA	NA		2 (9%)	2 (7%)		13 (29%)	4 (10%)	
More than 15 years	NA	NA		15 (65%)	26 (87%)		19 (42%)	31 (74%)	
Missing	NA	NA		0 (0%)	1 (3%)		5 (11%)	3 (7%)	

* denotes significant difference with a *P*-value < 0.05

^a^*p*-values are the result of the Student’s t test for age, and chi-square test for the rest categories to measure the difference between Birmingham and Wilcox County in 2016, 2018, and 2019, respectively

^b^Includes Asian, Alaskan Native or American Indian, White or European, Hispanic or Latino, or mixed race

^c^Includes “Excellent”, “Good”, and “Fair”

### Environmental health priorities and responsible parties identified in 2016 focus groups

A total of 83% participants in Birmingham and 12% participants in Wilcox County believed urban areas had worse environmental problems than rural areas (*p-*value 6.3E-13). Most participants (88% participants in Birmingham and 84% participants in Wilcox County, p-value 0.56) believed their communities did not receive its fair share of state and local resources devoted to environmental health problems.

Table 2 reports the environmental priority findings in the six focus groups in Birmingham. All six groups agreed that the main priority was abandoned and unmaintained houses: “*All you have to do is ride through to see. It is a disgrace. Just driving through it’s so grown up (overgrown) that you can’t even see the house.*” Groups mentioned many health concerns they believed were exacerbated by abandoned housing and overgrown lots including general health (2 groups), carbon monoxide, cough, mold, and infectious diseases (1 group). For the reason(s) this issue persists, five groups brought up that authorities were unresponsive or they did not follow through, four groups discussed government maintenance was limited and slow, and four groups believed money was an issue. “*We’ve been going on 15 years trying to get something going with our councilor. You couldn’t get nothing.*” Five groups identified Birmingham City Council as the top responsible party. Solutions were proposed by participants. More individuals attending city county meetings (4 groups), contacting government agencies and reporting issues (4 groups), and asking for government patrol of abandoned houses and additional maintenance of properties (3 groups) were top suggested short-term solutions. For long-term solutions, ideas included greater participation in community and neighborhood meetings (2 groups): *“(You) Gotta go out there and see. And if you don’t go out there and participate then you’ll never see.*”, buy or mortgage abandoned houses/lots (2 groups), community hold authorities accountable (2 groups), and government confiscates, tears down (5 groups) and transfer the ownership of abandoned houses for better maintenance (4 groups).

**Table 2:** Themes on abandoning housing topics in Birmingham, Alabama 2016 focus groups

Topics		Themes
Reason(s) persist		Authorities are unresponsive or they do not follow through (5 groups)
		Government maintenance is limited/slow (4 groups)
		Money issues (4 groups)
		Problems involved in buying/selling/tearing down (3 groups)
		Abandon house owners do not pay taxes (2 groups)
		Unequitable distribution of government resources, wealthy areas rezoning poor streets to Birmingham (2 groups)
		Owners neglect maintenance (1 group)
		Lack of specifying maintenance instructions for owners (1 group)
		Abandoned houses are not government priority (1 group)
		Issues with outsiders buying and selling drugs in neighborhoods (1 group)
		Need to communicate with government, not just neighbors (1 group)
		Population decreases (1 group)
		Racism (1 group)
		Difficulty attending city council meetings (1 group)
Solutions at individual level	Long-term	Participate in the community/neighborhood (2 groups)
		Buy or mortgage abandon houses/lots (2 groups)
		Rehab nearby abandon lots/ houses (1 group)
	Short-term	Attend city council meetings (4 groups)
		Contact government and report issues (4 groups)
		Individuals maintain nearby abandon lots/ houses (2 groups)
		Pressure authorities to act (1 group)
		Curfew for kids (1 group)
		Sell the properties (1 group)
		Specify whom to contact about the abandoned home (1 group)
Solutions at neighborhood level	Long-term	Hold authorities accountable (2 groups)
		Community support city council representative (1 group)
		Engage community (1 group)
		Neighborhoods work together to address issue, establish a community residents council (1 group)
	Short-term	Community work together to clean up neighborhood (1 group)
		Raise awareness (1 group)
		Community council inform government about residents’ concerns (1 group)
Solutions at government level	Long-term	Government confiscates, tears down abandoned houses (5 groups)
		Transfer ownership to people who will maintain them (4 groups)
		Build new homes or homeless shelter after tearing down, build new parks for kids in abandoned lots (4 groups)
		Engage community (2 groups)
		Address abandoned houses to also address crime (1 group)
		Provide proper funding (1 group)
		Hire more government personnel to maintain/tear down (1 group)
		City and county governments work together (1 group)
		Adopt anti-blight ordinance (1 group)
	Short-term	Patrol abandoned houses (3 groups)
		City aids with maintenance (3 groups)
		Charge penalties for lack of maintenance (2 groups)
		Inform residents what is being done (1 group)
		Investigating ownership of abandoned houses (1 group)
		City oversees abandoned houses (1 group)

Table 3 reports the results in the six focus groups in Wilcox County. In Wilcox County, the focus groups focused on the priority issue of drinking water access and quality with some discussion on sewage and septic issues. All six groups were concerned about the smell, look and taste of water. Five groups were concerned about the lack of water access and water-borne diseases. Primary health concerns that arose in discussion included cancer (5 groups), obesity (1 group), and infectious agents associated with poor sanitation (1 group). “*And it’s just awful. It’s awful because it makes your yard smell. It makes everything smell like septic.*” Four groups believed this issue has persisted because of unresponsiveness from authorities, particularly the county commissioners, and three groups suggested the lack of knowledge, information, and resources led to problem persistence. As for individual level solutions, participants suggested pressuring and reaching out to local government representatives (5 groups), attending county commission meetings and water board meetings (4 groups), and avoiding the use of county water (e.g., use bottled water) (2 groups). At the neighborhood level, participants mentioned organizing petitions and protesting would raise awareness (5 groups) and building trust, uniting, and engaging communities and organizing community meetings (3 groups). At the government level, they saw providing grants for installation and improvement of septic systems (5 groups) as well as testing water and distributing findings (3 groups), as important next steps to solve this issue.

**Table 3:** Themes on water quality in Wilcox County, Alabama in 2016 focus groups

Topics	Themes
Reason(s) persist	Unresponsive authorities and county commission issues (4 groups)
	Lack of knowledge/information/resources (3 groups)
	Money issues (2 groups)
	Residents are disenfranchised, low voter turnout (2 groups)
	Water pipe located far away (1 group)
	Distrust of government (1 group)
	Personal issues (1 group)
	Racism (1 group)
Solutions at individual level	Pressure/contact the government (5 groups)
	Attend county commission meetings, water board meetings (4 groups)
	Avoid using county water, use bottled water (2 groups)
	Boil water or use distilleries (2 groups)
	Collect evidence of racism/unequal treatment (1 group)
	Pray (1 group)
Solutions at community level	Petition/protest/raise awareness (5 groups)
	Build trust in/unite/engage the community (3 groups)
	Organize community meetings (3 groups)
	Provide resources, training, education (2 groups)
	Get the news involved (1 group)
	Provide general fund (1 group)
Solutions at government level	Provide grants for installing and improving septic systems, be transparent on how money is spent (5 groups)
	Test water and distribute findings (3 groups)
	County commission act (2 groups)
	Create water authority (2 groups)
	Improve septic system, fix water pipes (2 groups)
	Come up with solution (1 group)
	Have commission meetings at convenient times (1 group)
	Stop over-chlorinating water (1 group)
	Work with local government (1 group)

### Environmental health priorities change over time

**Fig. 1:**
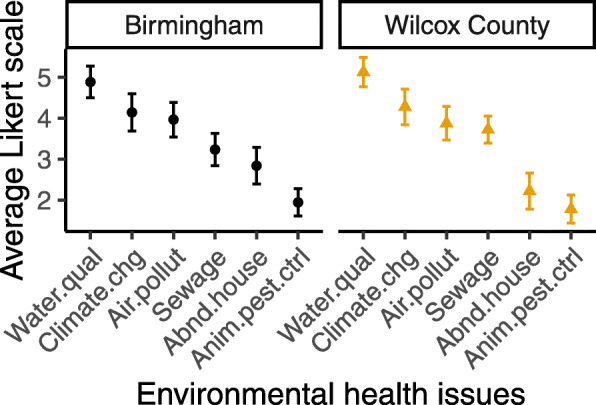
Mean Likert scale for environmental health issues in 2019 workshops. 95% confidence intervals were shown. Water.qual = water quality, climate.chg = climate change, Air.pollut = air pollution, Sewage = sewage and septic, Abnd.house = abandoned houses and lots, Anim.pest.ctrl = animal and pest control

Compared to our initial focus groups in 2012 [9] and our follow-up phone surveys in 2016 [11], and finally our focus groups in 2016 and workshops in 2018 and 2019 described herein, environmental health priorities changed over time in Birmingham, but stayed consistent in Wilcox County. In the follow-up workshops in 2018, 16 (70%) of participants in Birmingham agreed that abandoned housing was the primary environmental health priority while 21 (70%) of participants in Wilcox County agreed that drinking water and wastewater issues was the primary environmental health priority (Tables 4-5). However, in the 2019 follow-up workshops, participants from both locations ranked water quality as the No.1 environmental health priority (Fig. 1). Based on the median ranks, Wilcox participants ranked sewage and septic systems a higher priority compared with Birmingham participants (4.0 in Wilcox vs. 3.0 in Birmingham, Mann-Whitney test *p*-value 0.049) while they ranked abandoned houses/lots a lower priority (2.0 in Wilcox vs. 3.0 in Birmingham, Mann-Whitney test *p-*value 0.046).

**Table 4:** Follow-up survey results from workshops in 2018 in Birmingham, AL

Location	Birmingham
Participant number (%)	23 (100)
I am concerned about abandoned houses and overgrown lots because:	
Crime: drugs, sexual assault, vandalism, break-ins	16 (69.6)
Property values	4 (17.4)
Bad environment for youth in the community	2 (8.7)
Other (crime and property values)	1 (4.3)
Who do you think is most responsible for getting rid of abandoned houses and keeping up overgrown lots?	
Homeowners who move away or the family of deceased homeowners	10 (43.5)
Local government: city, mayor, city councilors	9 (39.1)
Residents in the neighborhood with the abandoned homes/overgrown lots	2 (8.7)
State government	1 (4.3)
Other (resident and local government):	1 (4.3)
What I can do personally to get rid of abandoned houses/ overgrown lots:	
Go to city council meetings	7 (30.4)
Go to neighborhood meetings	6 (26.1)
Call police when I see suspicious activity near an abandoned	5 (21.7)
Adopt a lot nearby and maintain it	2 (8.7)
Drive others to Neighborhood or city council meetings	1 (4.3)
Other (all of them)	1 (4.3)
Other(neighborhood and city council meeting)	1 (4.3)
What I would like to see community leaders do:	
Go to city council meetings and report back to community at	11 (47.8)
Communicate progress, plans, timelines	5 (21.7)
Organize community clean up days and recruit people to participate	3 (13.0)
Create a liaison position to communicate between the elected	2 (8.7)
Other (all of them)	2 (8.7)
What I would like to see state and local governing officials do (pick two):	
Devote more work crews tearing down abandoned houses, mowing overgrown lots, and towing abandoned cars	14 (60.9)
Provide incentives to build new businesses or new homes	6 (26.1)
Provide more police presence in the neighborhoods	5 (21.7)
Put signs up on houses/properties and advertise in the newspaper so potential buyers can know they are available for purchase	3 (13.0)
Reducing the waiting time to purchase houses/ properties	2 (8.7)
Devote more resources towards contacting previous owners	1 (4.3)
Remove the liens for back taxes faster to incentivize the Buy-	1 (4.3)
Other	1 (4.3)
In place of torn down houses, I would like to see:	
New houses by Habitat for Humanity	14 (60.9)
New housing for homeless	5 (21.7)
Community gardens or parks	3 (13.0)
New businesses	1 (4.3)
What one specific thing would you most like to see besides progress on the abandoned houses/overgrown lots issue? (Circle 1/ ONE)	
More police presence	8 (34.8)
A Community program and playground for kids	5 (21.7)
Other	3 (13.0)
High traffic roads paved	2 (8.7)
A program for mold removal from houses	2 (8.7)
A Senior Center within the community	2 (8.7)
Missing	1 (4.3)
Are abandoned houses and overgrown lots your most important 1/ ONE environmental health priority for your community?	
Yes	16 (69.6)
No	7 (30.4)
If no to previous question, what is your most important 1/ ONE environmental health priority for your community?	
Abandoned houses/lots	16 (69.6)
Crime and safety	2 (8.7)
Air pollution	1 (4.3)
Water quality	1 (4.3)
Other (road conditions, trash, and waste, work together)	3 (13.0)
Trusted information source	
TV	8 (34.8)
City Council meetings	6 (26.1)
Neighborhood meetings	5 (21.7)
Phone calls or conversations with community leaders	5 (21.7)
Newspaper	4 (17.4)
Radio	3 (13.0)
Health Department	2 (8.7)
Neighbors	2 (8.7)
Other	1 (4.3)

**Table 5:** Follow-up survey results from workshops in 2018 in Wilcox County, AL

Location	Wilcox County
Participant number (%)	30 (100)
Several issues relating to drinking water and wastewater have been brought up as issues in this community. Of these 3, what one issue is MOST important to you?	
Quality of the drinking water	17 (56.7)
Access to drinking water	8 (26.7)
Access to wastewater treatment(e.g., hookup to municipal sewage line or community or private septic system)	5 (16.7)
I am concerned about access and quality of the drinking water and wastewater treatment issues because: (Circle your 1/ ONE top reason)	
Taste, color, and smell are concerning.	23 (76.7)
Digging wells is too expensive	2 (6.7)
Installing private septic systems is too expensive	2 (6.7)
Other (use city water, health impact)	2 (6.7)
Missing	1 (3.3)
Who do you think is MOST responsible for giving people access to drinking water in their homes? (Circle your 1/ ONE top choice)	
Local Government like the County Commissioners	13 (43.3)
Water Board or Water Authority	12 (40.0)
Homeowners	2 (6.7)
Health Department or Extension Service	1 (3.3)
Homeowners and water board	1 (3.3)
Local government and water board	1 (3.3)
Who do you think is MOST responsible for ensuring quality of the drinking water in their homes?	
Local Government like the County Commissioners	13 (43.3)
Water Board or Water Authority	10 (33.3)
State Government	2 (6.7)
Health Department or Extension Service	1 (3.3)
Homeowners	1 (3.3)
Other (Homeowners and water board)	2 (6.7)
Missing	1 (3.3)
What I can do personally to ensure my drinking water is safe: (Circle 1/ ONE top choice)	
Go to Water Board meetings and County Commissioner Meetings	20 (66.7)
Read the annual statement from the water company on quality	7 (23.3)
Talk to my community at church to be united on the issue	1 (3.3)
Other (Don’t drink it)	1 (3.3)
Missing	1 (3.3)
What I would like to see community leaders do to address the water issues: (Circle 1/ ONE top choice)	
Write grant proposals for money to either fix the problem or get money to incentivize more people to participate in the process	15 (50)
Hold a local meeting for the community to be more informed and united	10 (33.3)
Bring media attention to the issue	2 (6.7)
Focus on getting young people involved	1 (3.3)
Missing	2 (6.7)
What I would like to see state and local governing officials do: (Circle TWO top priorities)	
Put more resources towards water lines, wells, and wastewater treatment	15 (50)
Evaluate whether the pipes are safe or need to be replaced	11 (36.7)
Have independent people test the water and communicate	7 (23.3)
Cancer Cluster Study to see if people in this area have more	6 (20.0)
Seek assistance from other nearby communities to share	2 (6.7)
Communicate where current resources are going	1 (3.3)
Set aside differences and come to an agreement on a specific	3 (3.3)
Missing	1 (3.3)
What 1/one specific thing would you most like to see besides progress on the drinking water and wastewater issues? (Circle 1/ ONE)	
A community program for kids	9 (30.0)
An informative program for understanding healthcare options and process	8 (26.7)
Other (better roads; work with local and state board; community meeting with local information; illiteracy program; need more than just one option):	5 (16.7)
Bright streetlights, roadside signs, and State Highway	4 (13.3)
More animal control	3 (10)
Missing	1 (3.3)
Are drinking water and wastewater issues your most important 1/ ONE environmental health priority for your community?	
Yes	21 (70.0)
No	7 (23.3)
Missing	2 (6.7)
If no to previous question, what is your most important 1/one environmental health priority for your community?	
Drinking water and wastewater	21 (70.0)
Crime and drugs	2 (6.7)
Clean streets trash waste spill	4 (13.3)
Road	1 (3.3)
Health outcomes	1 (3.3)
Program for kids	1 (3.3)
Trusted information source	
TV	10 (33.3)
Radio	10 (33.3)
County commission meetings	8 (26.7)
Health Department	7 (23.3)
Monthly water board meetings	6 (20.0)
statements mailed from the water Company	4 (13.3)
Phone calls or conversations with community leaders	3 (10.0)
Newspaper	3 (10.0)
State and local leaders	3 (10.0)
Other	1 (3.3)
Missing	1 (3.3)

In the 2018 follow-up workshops, more than half of the participants believed state and local resources were not fairly distributed to communities to address environmental health problems (87% in Birmingham and 67% in Wilcox, *p-*value 0.59), and both communities suggested a lack of leadership at the local level was the top reason behind this unfair distribution. In these workshops, Birmingham participants built from the 2016 focus group results described above, stating they would like to see neighborhood leaders attend city council meetings and report back to neighborhood residents (48% participants), and communicate progress, plans and timelines on addressing abandoned housing and vacant lots in their neighborhoods (22% participants). Birmingham participants stated that more state and local government resources should be devoted to hiring more work crews to tear down abandoned houses, mow overgrown lots (61% participants), provide incentives to build new business or new homes (26% participants), and provide more police presence (22% participants). Wilcox county participants in the 2018 follow-up workshops suggested community leaders should write grant proposals for money to fix the septic issues (50% participants) and hold local meetings to inform and unite residents (33% participants). They would also like to see state and local governing officials put more resources towards water lines, wells, and wastewater treatment (50% participants), and evaluate whether the pipes are safe or need to be replaced (37% participants) (Tables 4-5).

### Sources of trusted information

The most trusted sources of information were news on television (TV), city council and city council representatives, and word-of-mouth in Birmingham, all of which were mentioned by four focus groups. Radio (5 groups), news on TV (4 groups), and word-of-mouth (3 groups) were the most trusted source of information in Wilcox County. One group in Wilcox County reported trust in local government, but not the county commission or mayor. In contrast, Birmingham focus groups frequently cited the government as a trusted source of information, in particular their city council and city council representative. Of the two Birmingham focus groups that did not cite the government as a trusted source of information, one did not mention any sources of trusted information and the other only cited the news and newspaper. While the Birmingham focus groups never mentioned distrusted sources of information, one group did say there was a lack of a trusted source of information. Similar to 2016 focus group results, in the 2018 follow-up workshops, Birmingham survey participants identified TV, city council meetings and neighborhood meetings, and conversations with community leaders as the most trusted information sources, while Wilcox County participants identified TV, radio, and county commission meetings as the most trusted information sources (Tables 4-5).

## Discussion

The ENACT community-academic partnership has been engaging with residents in Birmingham AL and Wilcox County AL since 2010 to understand environmental health priorities through focus groups, phone surveys, written surveys and workshops [9, 11]. Here we present results from our most recent focus groups and workshops that clarified priorities, possible solutions, responsibly parties, and sources of trusted information on priority issues. We found that the environmental health priorities of abandoned houses in Birmingham and drinking water issues in Wilcox County in the focus groups were consistent with our previous findings [9].

The results suggest that participants saw local government non-responsiveness as the top reason for issues with abandoned housing persisting (5 of 6 focus groups), while also acknowledging government actions as the most promising solutions in addressing the abandoned housing issue in Birmingham (Table 2). In the 2018 workshops, 39% participants in Birmingham reported they believed local government (city, mayor, city councils) were most responsible for getting rid of abandoned houses (Table 4), showing consistency over time. In Wilcox County, five out of six focus groups discussed a range of solutions at the individual level, community level and government levels, which suggests that participants in Wilcox County see involving all stakeholders to tackle the water and sanitation problems is most promising.

The results, together with identified persistence reasons and potential solutions at the individual, community, and government levels may serve as evidence-based tools for identifying actions in the future. Follow-up workshops not only provided the opportunity to examine whether the identified environmental health priorities change over time but also serve as the events where research results were disseminated back to residents. As environmental health is a dynamic and evolving field, the environmental issues in Birmingham and Wilcox County communities can change over time. For example, Birmingham City Council programs initiated between 2016 and 2019 [41, 42] could have contributed to reducing residents’ concerns over abandoned housing and vacant lots in 2019. Alternatively, the number of Public Water Systems with any violation dropped from 117 in 2013, to 61 in 2018, to 110 in year 2020 in Alabama [43], suggesting water quality issues have not changed.

Knowing from what sources people get trusted information on environmental health issues can shed light on why people are concerned about particular risks. Results showed that both communities trusted news on TV and word-of-mouth, and Birmingham groups trusted city council and Wilcox groups trusted radio programs. We also found a lack of trust in government in Wilcox groups. The results are consistent with a similar study surveying rural residents in El Paso, Texas where 54 and 46% participants had high confidence in television and radio, respectively, and the participants had low confidence in the government as a source of information [44]. As suggested by Byrd et al. (1997), the way that risk is portrayed by the media and the selection of stories may impact people’s perception of environmental health priorities [44]. Knowing the trusted information source may help community leaders monitor emerging or ongoing environmental health priority topics as well as use these sources to involve more residents, spread updates of meetings and policies, and disseminate evidence-based solutions. The names of specific TV programs or radios stations where participants get trusted information can be collected in future studies.

There are some limitations in the study. Bias may have been introduced with the use of nonprobability sampling methods to recruit focus group and workshop participants; however similar participant demographics in Birmingham and Wilcox County reduced potential bias when comparing results between the two locations, as studies have shown that gender, race, and culture are primary influences on risk perception [45]. As is common in health studies [46], the results presented herein reflect higher participation rates of women in both Birmingham and Wilcox County events, therefore male perspectives, if different, are underrepresented. Focus group participants may have refrained from bringing up issues in front of other community members or respected community leaders. However, in the focus group setting, participants could add to others’ responses to clarify issues and direct discussion in a meaningful way. Participants may also feel empowered by voicing their opinions and insights with other residents. There were some technical challenges in understanding some of the audio recordings, specifically distinguishing individual speakers within the group. This technical challenge coupled with high agreement within each group led to group-wise comparisons instead of individual counts as reported in previous focus groups [9]. As noted in the methods, the coding groups were not mutually exclusive, and topics could be counted multiple times, which is common to focus group analysis methodologies [38]. There was a higher percent of returning participants in Wilcox compared to Birmingham, which may have contributed to the result that drinking water quality was consistently the number one environmental priority in Wilcox County, however we do note that a randomly sampled phone survey we conducted also identified water quality as a top priority [11] .

## Conclusions

Focus groups conducted in 2016 reaffirmed the identified environmental health priorities in 2012 focus groups, in both urban and rural communities. The top environmental health priority remained water quality and sewage treatment in the rural community in 2018 and 2019 surveys but switched from abandoned houses to water quality in the urban community in 2019. Participants identified ways to support the community in identifying and enacting solutions to their environmental concerns, which can be useful for community leaders to make future changes to address the problems.

## Supplementary Information

**Additional file 1.** Semi-structured discussion guide for focus groups and activities for workshops.

**Additional file 2.** Transcript coding tree to identify persistence reasons, responsible parties, solutions, and source of trusted information.

## Data Availability

The dataset generated and analyzed during the current study are not publicly available due to the identifiable audio recordings, identifiable demographic information and questionnaires from participants. De-identified and aggregated data can be obtained by request to the corresponding author, Julia Gohlke, at jgohlke@vt.edu.

## Abbreviations

AL: Alabama
FoWE: Friends of West End
WCACHIL: West Central Alabama Community Health Improvement League
IRR: Interrater reliability rate
TV: Television
