# Supplementary material for: A community-engaged approach to understanding environmental health concerns and solutions in urban and rural communities
Source: BMC Public Health. 2021 Sep 24;21:1738. doi: 10.1186/s12889-021-11799-1 (PMC8464125; doi:10.1186/s12889-021-11799-1)
Supplement: Supplementary file 1 — Additional file 1. Semi-structured discussion guide for focus groups and activities for workshops. [file 12889_2021_11799_MOESM1_ESM.pdf]

Additional files of

A community-engaged approach to understanding environmental health concerns and solutions in urban and rural communities

Suwei Wang[1,2], Molly B. Richardson[3], Mary B. Evans[4], Ethel Johnson[5], Sheryl Threadgill-Matthews[5], Sheila Tyson[6], Katherine L.White[4], Julia M. Gohlke[2*]

[1] Translational Biology, Medicine, and Health Program, Virginia Polytechnic Institute and State University, Blacksburg, VA 24061
[2] Department of Population Health Sciences, Virginia Polytechnic Institute and State University, Blacksburg, VA 24061
[3] Division of Preventive Medicine, School of Medicine, University of Alabama at Birmingham, Birmingham, AL 35233
[4] Center for the Study of Community Health, University of Alabama at Birmingham, Birmingham, AL 35233
[5] West Central Alabama Community Health Improvement League, Camden, AL 36726
[6] Friends of West End, Birmingham, AL 35228

*Corresponding author:
Julia Gohlke, PhD
Associate Professor
Department of Population Health Sciences
VA-MD College of Veterinary Medicine
Virginia Polytechnic Institute and State University
205 Duck Pond Drive
Blacksburg, VA 24061-0395
jgohlke@vt.edu

Additional file 1. Semi-structured discussion guide for focus groups and activities for workshops.

Focus groups in 2016
Is water access still the number 1 priority? (Wilcox County). Are abandoned houses, abandoned or overgrown lots still the number 1 priority? (Birmingham)
Why does it persist?
What are the potential short- or long-term solutions?
What are the responsible parties?
What are the sources of trusted information?
What are the environmental health consequences you are concerned with the most?
Are you concerned with other priorities?
**Workshop in 2018**
River of life introduction
Brief presentation on previous focus group and current status by community leaders
Research results from air pollution monitoring and temperature monitoring from academic researchers
Small group discussion on 'keep the river flowing: hopes for the future'.
Large group discussion and closing remarks
**Workshop in 2019**
Climate change and health by academic researcher
Table discussion and message development on climate change and health
Presentation of table discussion results
Large group discussion and closing remarks
