# Supplementary material for: A community-engaged approach to understanding environmental health concerns and solutions in urban and rural communities
Source: BMC Public Health. 2021 Sep 24;21:1738. doi: 10.1186/s12889-021-11799-1 (PMC8464125; doi:10.1186/s12889-021-11799-1)
Supplement: Supplementary file 2 — Additional file 2. Transcript coding tree to identify persistence reasons, responsible parties, solutions, and source of trusted information. [file 12889_2021_11799_MOESM2_ESM.pdf]

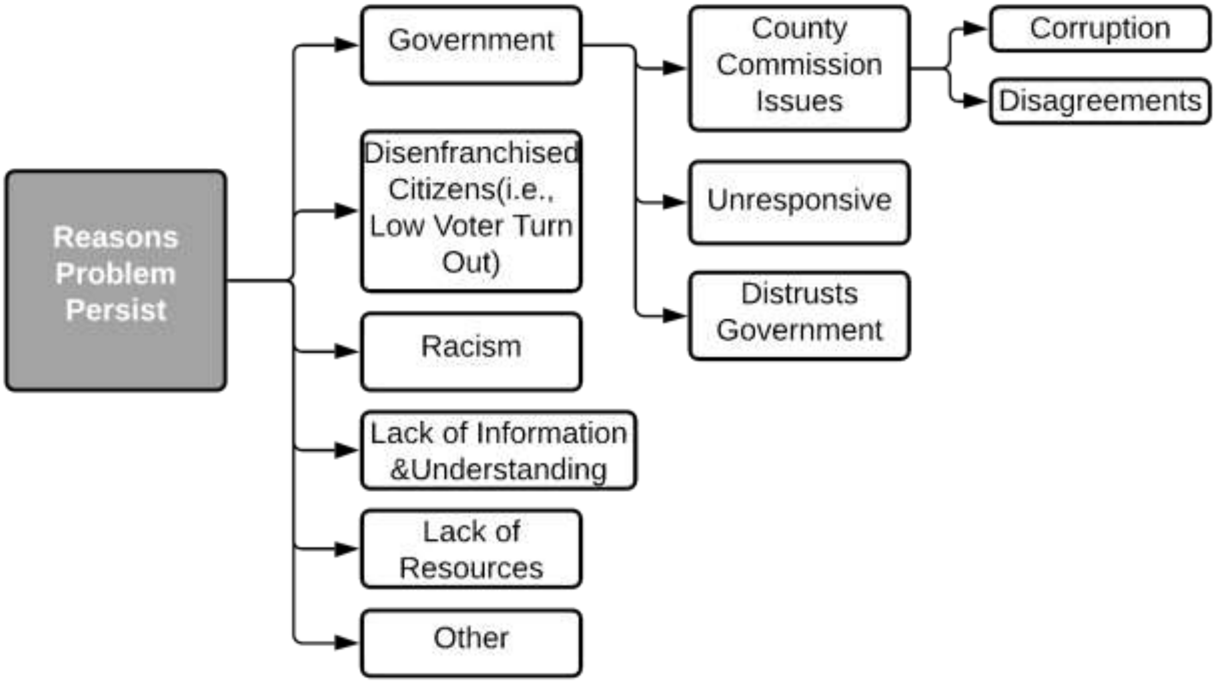

Additional file 2. Transcript coding tree to identify persistence reasons, responsible parties, solutions, and source of trusted information. Phase 1 coding in gray background color, phase 2 coding in white background color.

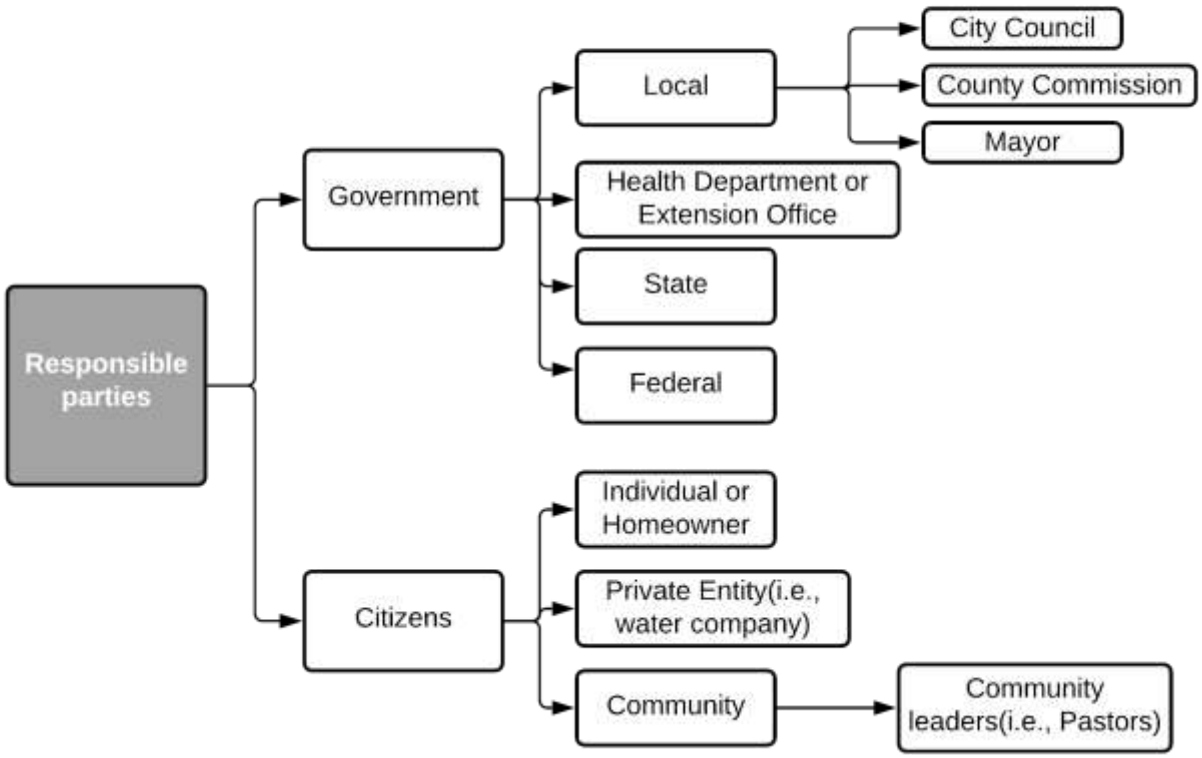

Additional file 2 continued. Transcript coding tree to identify persistence reasons, responsible parties, solutions, and source of trusted information. Phase 1 coding in gray background color, phase 2 coding in white background color.

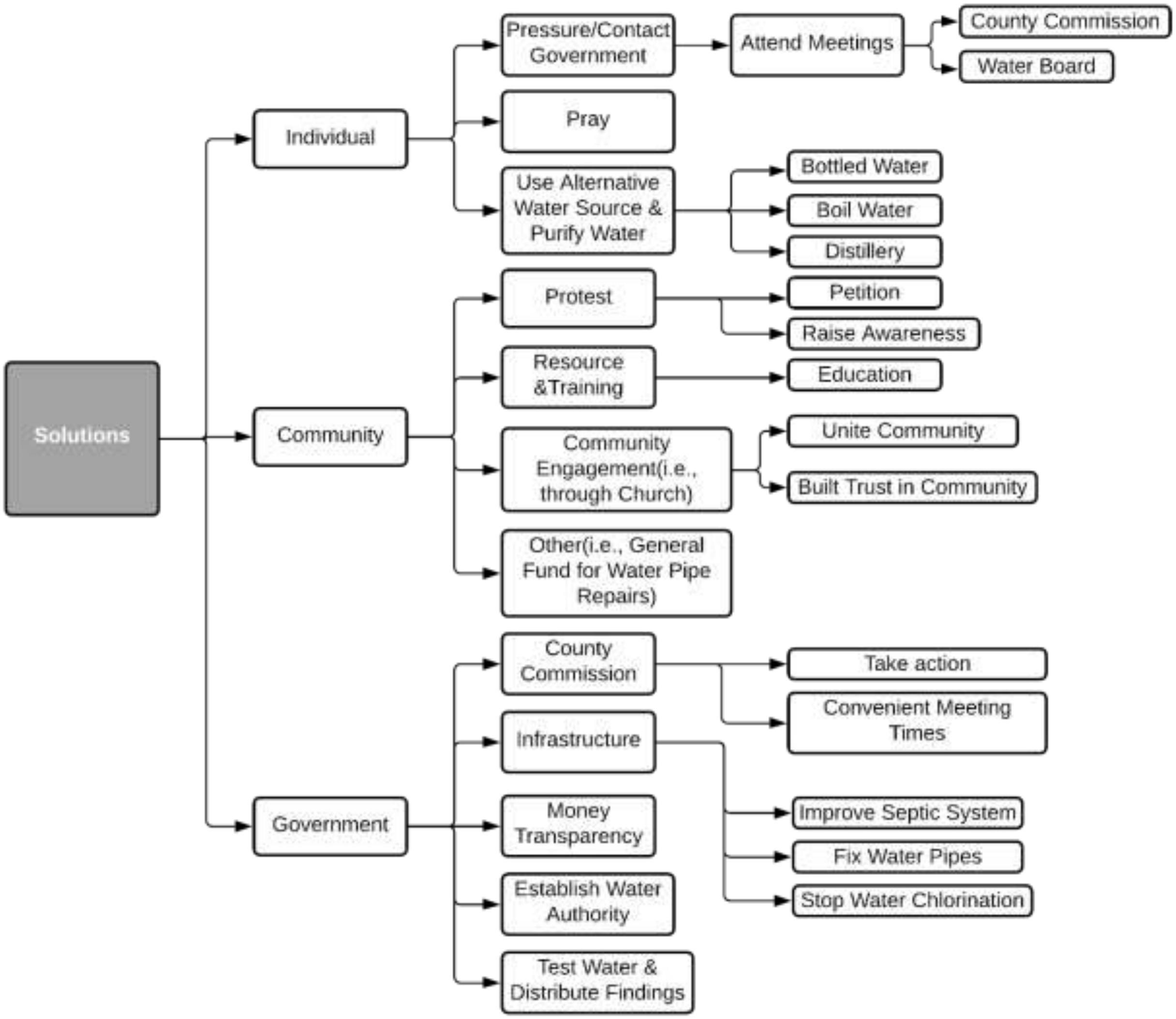

Additional file 2 continued. Transcript coding tree to identify persistence reasons, responsible parties, solutions, and source of trusted information. Phase 1 coding in gray background color, phase 2 coding in white background color.

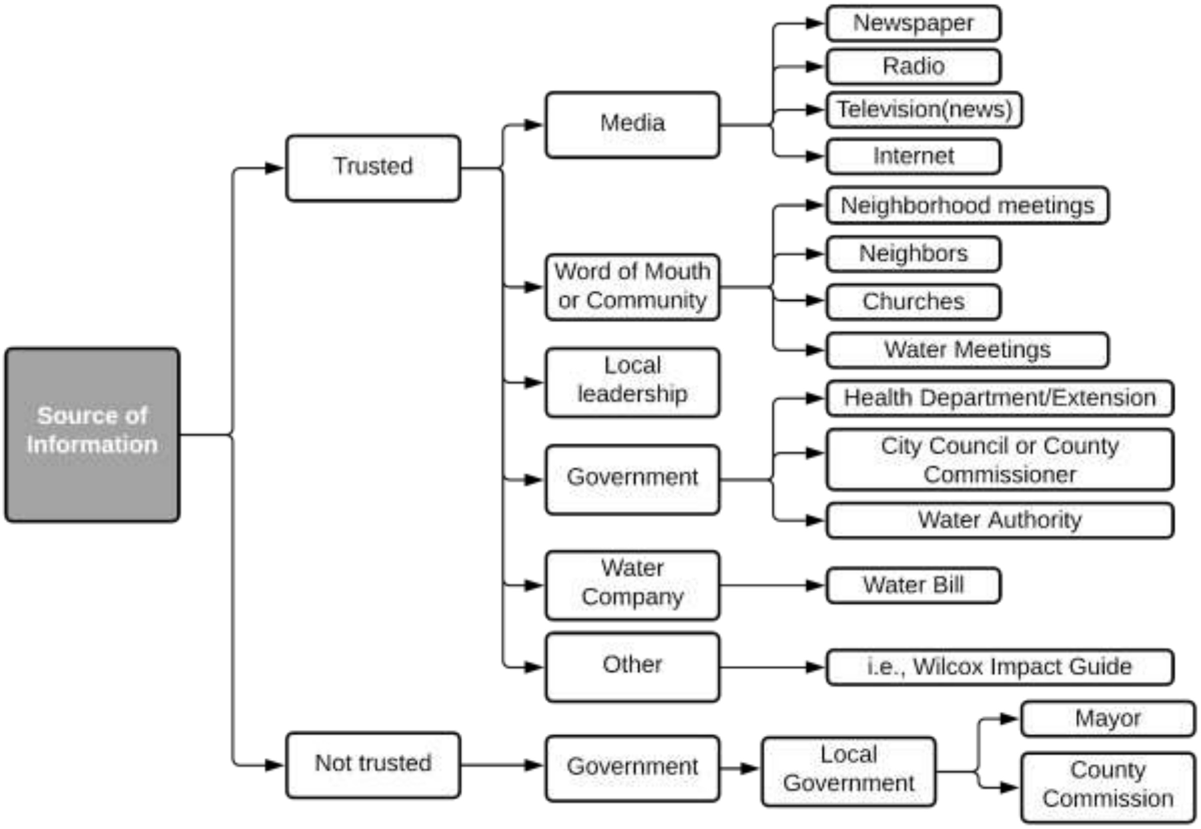

Additional file 2 continued. Transcript coding tree to identify persistence reasons, responsible parties, solutions, and source of trusted information. Phase 1 coding in gray background color, phase 2 coding in white background color.

Additional file 3. Themes on abandoning housing topics with verbatim in Birmingham, Alabama 2016 focus groups.

Topics	Themes	Verbatim example
Reason(s) persist	Authorities are unresponsive or they do not follow through (5 groups)	“We’ve been going on 15 years trying to get something going with our councilor. You couldn’t get nothing.”
	Government maintenance is limited/slow (4 groups)	"They ain’t doing nothing but cutting the grass."
	Money issues (4 groups)	“People don’t have the money.”
	Problems involved in buying/selling/tearing down (3 groups)	"Problem of why there are so many houses. Once you apply you still have to wait so many months to tear them down. Or purchase them. That’s why maybe some of them haven’t been torn down. "
	Abandon house owners do not pay taxes (2 groups)	“nobody’s paying taxes on these properties”
	Unequitable distribution of government resources, wealthy areas rezoning poor streets to Birmingham (2 groups)	“Like I said they address the communities with the money.”
	Owners neglect maintenance (1 group)	“They (owners) don’t care. They’re going to throw their trash out the window.”
	Lack of specifying maintenance instructions for owners (1 group)	“We kept reporting it and reporting it and finally they came and cut the grass, but now it’s just how it was (implying overgrown again). It’s just like you said—they’re not consistent (with maintenance).”
	Abandoned houses are not government priority (1 group)	"They’re not doing nothing with them.”
	Issues with outsiders buying and selling drugs in neighborhoods (1 group)	“They cause a lot of drugs.”
	Need to communicate with government, not just neighbors (1 group)	“They have town hall meetings that are really good to go to. Then you can voice your opinion about anything. Anything you want to see them do. I told them about some things.” "communication Between the neighborhood and the city."
	Population decreases (1 group)	"The kids moved out and now we’ve got 2 empty houses sitting behind us. "
	Racism (1 group)	"They (city or county officials) don’t come and do that much for our neighborhood. I tell you if you go over the mountain to where the white people live, and everything is nice. "

		Difficulty attending city council meetings (1 group)	"They got to make city hall more accessible to handicapped people. "
Solutions at individual level	Long-term	Participate in the community/neighborhood (2 groups)	"You've got to participate. "
		Buy or mortgage abandon houses/lots (2 groups)	"They could have taken a second mortgage on it. "
		Rehab nearby abandon lots/ houses (1 group)	"A lot of them could be rehabbed because I know a lot of people in the other cities that do that."
	Short-term	Attend city council meetings (4 groups)	"Go to the city council meetings"
		Contact government and report issues (4 groups)	"Contact XX (an official's name) office and let them know what's going on. Because we have to be the eyes and ears of the community because they can't be everywhere. Just let them know"
		Individuals maintain nearby abandon lots/ houses (2 groups)	"You've got to keep the lot clean, keep the house repaired, keep current on your mortgage, taxes, anything like that" Makes me want to take a broom and get there to sweep.
		Pressure authorities to act (1 group)	"We need to be sitting down there in the audience so that we can voice our opinion to them."
		Curfew for kids (1 group)	"There's supposed to be a curfew for kids under 17. At a certain time they're supposed to be at their house. "
		Sell the properties (1 group)	"I say after a certain time, put it up for sale."
		Specify whom to contact about the abandoned home (1 group)	"You need a will made so that if anything were to happen, they will know who to contact. "
Solutions at neighborhood level	Long-term	Hold authorities accountable (2 groups)	"You've got to pay up in the thousands to cut a tree down. That's the act of the lord. I feel like the city shouldn't involve themselves. We didn't plan the trees. We need to put a law enforcement on that. Somebody to go around and trim the trees."
		Community support city council representative (1 group)	"We have people here. Not only XX (an official's name), but representatives from the different departments like human resources."
		Engage community (1 group)	"But I'm saying you can call and ask each other to come here. We can call, we can call other people to meet us so that we can voice our opinions. Everybody needs to know when the city council meeting is."

		Neighborhoods work together to address issue, establish a community residents council (1 group)	"You can call and ask each other to come here. We can call, we can call other people to meet us so that we can voice our opinions. Everybody needs to know when the city council meeting is." "Your neighborhood gets together and agrees on a day to clean your neighborhood up. Walk through all the alleys. And the people who are working to pay a fine, let them come out to help."
	Short-term	Community work together to clean up neighborhood (1 group)	"Some of us need to come together and get whatever we can."
		Raise awareness (1 group)	"We can call and ask each other to come here. We can call, we can call other people to meet us so that we can voice our opinions. Everybody needs to know when the city council meeting is."
		Community council inform government about residents' concerns (1 group)	"If we see a problem, we need to address it. And find the proper person to address it too. And that's our council person."
Solutions at government level	Long-term	Government confiscates, tears down abandoned houses (5 groups)	"Knock down the old buildings and put new ones up"
		Transfer ownership to people who will maintain them (4 groups)	"Transferring ownership to people who will maintain the property, either maintain the property or demolish the property and do something else with it. "
		Build new homes or homeless shelter after tearing down, build new parks for kids in abandoned lots (4 groups)	"Give them to the homeless so that the homeless have somewhere to go."
		Engage community (2 groups)	"It needs the support of all of the people. Not just one or two. Everybody needs to work together on this in order to make it work."
		Address abandoned houses to also address crime (1 group)	"But if they would do something about the abandoned houses and all this stuff, there wouldn't be as much crime. Because they have a place to run to hide."
		Provide proper funding (1 group)	"There should be some kind of program where people can redeem those houses. To come up with the money to fix them up instead of tearing them down."
		Hire more government personnel to maintain/tear down (1 group)	"That would be good if you got the responsible people to take on maintaining that. That would be good for neighborhood."
		City and county governments work together (1 group)	"So Jefferson County and the City of Birmingham need to get together and talk. Whoever that district person is for Jefferson County needs to get together with the county people."

		Adopt anti-blight ordinance (1 group)	"They call them anti-blight ordinance. In other words you've got to keep the lot clean, keep the house repaired, keep current on your mortgage, taxes, anything like that"
	Short-term	Patrol abandoned houses (3 groups)	"We need more police patrol in the targeted areas like this. With West End as the targeted area."
		City aids with maintenance (3 groups)	"The city will give assistance to improve your house—put rubs on, put an addition, but it's a long drawn out process. He'll hire more people to work and they'll work like 10 hours every day. Get the work done."
		Charge penalties for lack of maintenance (2 groups)	"And if they warn them 3 times that they need to go out and cut the grass, they need to be fined."
		Inform residents what is being done (1 group)	"We're reaching out to the city. We want the city to reach out back to us!" "Send a mailout"
		Investigating ownership of abandoned houses (1 group)	"Tracking down the original owners. And then they have to clear the property completely before they can let people purchase it."
		City oversees abandoned houses (1 group)	"That would be good if you got the responsible people (city and mayor) to take on maintaining that. They should get a law passed that says if nobody answers these letters that we send to them (owners) that after so long they just lose out on them."

Additional file 4. Themes on abandoning housing topics with verbatim in Wilcox County, Alabama 2016 focus groups.

Topics	Themes	Verbatim example
Reason(s) persist	Unresponsive authorities and county commission issues (4 groups)	"Those in charge don't want to listen to us. They hear it, but don't."
	Lack of knowledge/information/resources (3 groups)	"Training so I can be aware of what am I looking for. What I need to…What I need to know about it. What I need to know first so that I can ask for the resources."
	Money issues (2 groups)	"It costs so much money and the people don't want to pay for it. Or they can't afford to pay for it."
	Residents are disenfranchised, low voter turnout (2 groups)	"I didn't hear anybody address it at the last meeting. But every time they address it, it's gets voted 3 -3. It can't go anywhere."
	Water pipe located far away (1 group)	"The pipe is so far from wherever [they need it to use it]. "
	Distrust of government (1 group)	"I don't think there is anybody we can trust."
	Personal issues (1 group)	"They can't see beyond each one's personal agenda.
	Racism (1 group)	"I think another issue is black and white in this county. Wilcox County do not work together. There is still a lot of racism going on in this county. And we'll never get in on it because there is white on this side and black on this side."
Solutions at individual level	Pressure/contact the government (5 groups)	"We have to go to and get the word out to those who may be not as involved. Like there are people at this table that we know. We need to go back and make it an issue. Pastor at the church. Get the information out."
	Attend county commission meetings, water board meetings (4 groups)	"They make an announcement. So that's how you know when there's going to be a meeting. It's open to the public. We can go to the meetings"
	Avoid using county water, use bottled water (2 groups)	"We go and buy bottled water."
	Boil water or use distilleries (2 groups)	"I don't drink from my faucet. And really for cooking or taking a bath, you need to boil that too."
	Collect evidence of racism/unequal treatment (1 group)	"Proof. Evidence. Evidence is a sound thing. Get us a written agreement. They like proof. Recording. Get your agreement saying hey can I record you? Write it and record it and bring it back and let me see it. That's evidence. That's written proof of what so they can see how this is at. And then get written evidence, proof, of what's going on then."

	Pray (1 group)	"We got to pray hard."
Solutions at community level	Petition/protest/raise awareness (5 groups)	"Get everybody sign a petition"
	Build trust in/unite/engage the community (3 groups)	"If you get your community to join in and get involved, you'll get a lot done. "
	Organize community meetings (3 groups)	"Let's have a community meeting to let them hear well their needs and what is going on. They'll take it to the governor, the highest belt."
	Provide resources, training, education (2 groups)	"Commissioners that need to make the agreement. We as a people need to put pressure on them. And especially those that are in that district"
	Get the news involved (1 group)	"Maybe they need to put it back on the news until they do something about it. Because that's the only way to get about it. Embarrassment"
	Provide general fund (1 group)	"Grant writing to get some funding for the community"
Solutions at government level	Provide grants for installing and improving septic systems, be transparent on how money is spent (5 groups)	"It's money. We don't know about it. So would you like better communication about where the money is going?"
	Test water and distribute findings (3 groups)	"(Government) To test and give accurate results of a test. "
	County commission act (2 groups)	"And I think we're looking for and what will really solve the problem is for the commissioners to come to an agreement to go on and make a decision and quit battling along amongst themselves."
	Create water authority (2 groups)	"Get a water authority in place, but they're having a hard time with having all 6 of the commissioners to get in agreement to vote to have the water authority."
	Improve septic system, fix water pipes (2 groups)	"In my community, the pipes need to be changed."
	Come up with solution (1 group)	"Try to come up with a solution."
	Have commission meetings at convenient times (1 group)	"I always try to find a way to be involved but I don't because of timing as far as attending the meetings."
	Stop over-chlorinating water (1 group)	"Too much chlorine in the water."
	Work with local government (1 group)	"Get in touch with your local government."

This is the end of this file.
